# Cross-sectional analysis comparing prefabricated titanium to individualized hybrid zirconia abutments for cemented zirconia based fixed dental prostheses: a critical concept assessment

**DOI:** 10.1186/s40729-024-00529-y

**Published:** 2024-03-18

**Authors:** Norbert Neckel, Josephine Pohl, Saskia Preissner, Oliver Wagendorf, Claudia Sachse, Kirstin Vach, Max Heiland, Susanne Nahles

**Affiliations:** 1 Department of Oral and Maxillofacial Surgery, Charité-Universitätsmedizin Berlin, Corporate Member of Freie Universität Berlin, Humboldt-Universität zu Berlin, and Berlin Institute of Health, Berlin, Germany; 2 Faculty of Medicine and Medical Center, Institute of Medical Biometry and Medical Statistics, University of Freiburg, Freiburg, Germany

**Keywords:** Individualized zirconia abutments, Prefabricated titanium abutments, Emergence angle, Emergence profile, Bone loss, Cemented zirconia fixed dental prostheses (FDP)

## Abstract

**Purpose:**

Despite the differences in material properties and shapes among the different types of prefabricated titanium (pTiA) and individualized hybrid zirconia abutments (ihZiA), the biological and clinical relevance of materials and construction features remains vague. Yet, individualized ihZiA are increasingly implemented into daily routine aiming to satisfy rising expectations. The objective was to compare these two types of abutments in fixed dental prostheses (FDP).

**Methods:**

This cross-sectional study examined 462 implants in 102 patients comparing pTiA (52 patients) to ihZiA (50 patients) for FDP. These different treatment regimens were evaluated in terms of peri-implant health, radiographic bone loss, and oral-health related quality of life (OH-QoL) with special consideration of abutment type and superstructure design.

**Results:**

ihZiA showed significantly different design features than prefabricated pTiA, but the annual bone loss in both groups did not.

Visible titanium in the esthetic zone negatively impacted OHIP 14 scores. The combination of an emergence angle (EA) of < 30° and a concave emergence profile (EP) as well as gingiva thickness (p = 0.002) at the time of the prosthetic restoration significantly improved the annual peri-implant bone loss, independently of the abutment type.

**Conclusion:**

ihZiA showed comparable results to pTiA. To optimize the long-term outcome, not just material alone but generating adequate soft tissue thickness, minimizing the EA, and applying a concave EP seem to be the most relevant factors. To improve OH-QoL, particular attention must be paid to the esthetic zone.

**Graphical Abstract:**

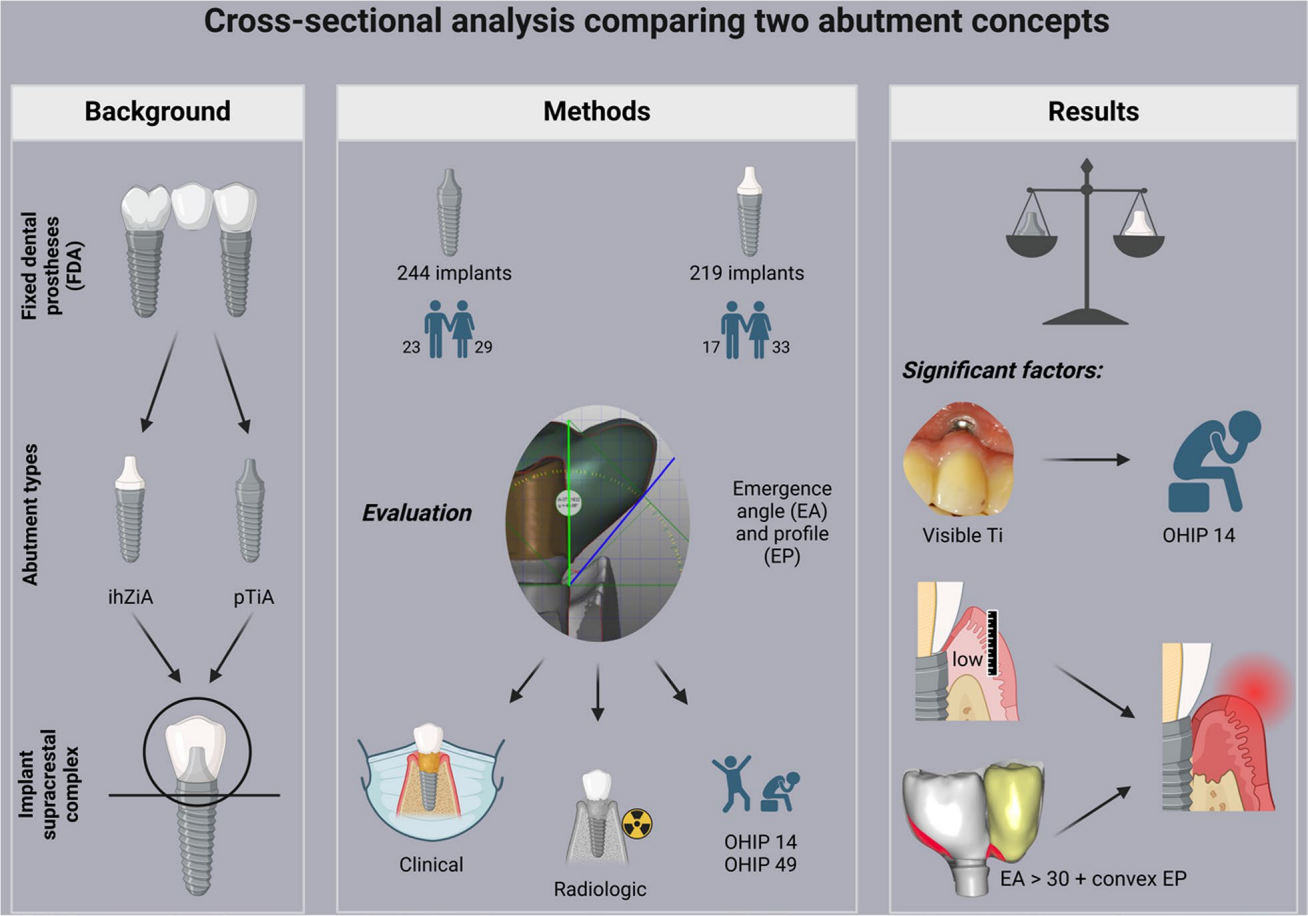

## Background

The expectations for implant-based restorations in terms of esthetics, function, and longevity are high, and various options for dental rehabilitation with implant-supported bridges exist. The main options are prefabricated, individualized, and two-piece abutments of different materials, which can support cement- or screw-retained fixed dental prostheses (FDP) [1, 2]. The proven abutment material titanium (Ti) shows good biological and mechanical properties to fulfill most needs [3]. However, its dark color is disadvantageous, especially in the esthetic zone. This led to the introduction of ceramic abutments, among which zirconia (Zi) abutments prevailed due to their excellent mechanical strength and titanium-like biocompatibility [1, 4]. Nevertheless, a brittle material susceptible to porcelain chipping or fractures due to long-term fatigue might be a problem in certain platform-switching systems [5]. This is important since implant abutment connections play a critical role in the long-term success of dental implants and can be considered the Achilles heel for bacterial contamination and marginal bone loss [6].

Another innovation increasingly implemented in implant dentistry via a digital workflow is the use of individualized abutments, which can be especially helpful in surgically difficult situations and restorations, including bridges with multiple abutment teeth [7]. Computer-aided design and computer-aided manufacturing (CAD/CAM) technologies provide the basis for patient- and implant location-specific abutments, which may help to reduce costs, eliminate inaccuracies, optimize individual anatomic features, and improve emergence profile (EP), gingival profile, and angulation [8, 9]. Prefabricated abutments, on the other hand, are proven to be safe, cheaper, and simple to use but fail to address interindividual differences [10].

Despite the many differences in material properties and shapes among titanium and zirconia abutments and prefabricated and individualized abutments, the actual biological and clinical relevance remains elusive [11]. This includes the actual impact of the materials applied as well as the abutment shape itself and the supracrestal complex. Nevertheless, the demand for patient specific solutions with the best possible biocompatibility and esthetic outcome is increasing. Consequently, individualized Zi based abutments (ihZiA) are increasingly implemented into dental practice. This is why we aimed to determine potential influencing factors in peri-implant tissue health in terms of material and abutment shape with regard to the design of the superstructure.

Therefore, this single-institution cross-sectional study documents the clinical and radiologic outcome of two treatment concepts, comparing a cohort of patients receiving prefabricated pTiA as a standard treatment with a cohort that was solely restored with individualized ihZiA and evaluate the outcome in terms of bone-loss and peri-implant tissue health, as well as oral-health related quality of life (OH-QoL). The primary hypothesis was that individualized ihZiA were not significantly different to pTiA concerning peri-implant marginal bone loss.

## Materials and methods

### Study protocol

All persons involved had provided their informed consent to their inclusion in this study. The ethics committee of the Charité—Universitätsmedizin Berlin, Germany (EA4/064/18) approved the study protocol and it conforms to the Declaration of Helsinki and the STROBE criteria for cross-sectional studies.

### Participants and setting

All patients (n = 102) included in the study had received implant-based cemented zirconia FDP from 2014 to 2016 mirroring the change in the general prosthetic concept from pTiA (until 2015) to hybrid zirconia abutments ihZiA (after 2015). The same surgeon and prosthodontist treated all patients (JP). Anamnestic data such as age, sex, opposing teeth/prosthetics, alcohol consumption, smoking, diabetes, medication, and the frequency of professional cleaning were documented, analyzed and accounted for as potential confounders. The patients were recruited in the context of the regular follow-up. All patients included had to be of full age (≥ 18 years) and gave informed consent to participate. They had to have been restored with either pTiA (Ø 5 mm with 4 or with a height of 7 mm, titanium grade 5 or 15° angled with a height of 7 mm, titanium grade 4, ICX medentis medical GmbH, Bad Neuenahr-Ahrweiler, Germany) or ihZiA (yttrium stabilized zirconium oxide, Prettau®-Zirconia, Zirkonzahn GmbH, Gais, Italy bonded to a titanium (grade 5) base of Ø 5,1 mm) supporting FDPs. Exclusion criteria were incapacity or unwillingness to give informed consent, immediately loaded implants, single-tooth implants, screw-retained FDP, nonattendance to follow-up and insufficient completion of the questionnaires. Using the respective questionnaires, patient OH-QoL was measured via the Oral Health Impact Profile-Germany (OHIP-G) 14 and 49 scores which were analyzed separately [12]. One hundred and sixty-five implants with pTiA restorations and 154 implants with ihZiA restorations were placed in the molar and premolar region, whereas 79 of the pTiA group implants and 64 of the ihZiA group implants were located in the anterior region from canine to canine.

### Surgical and prosthetic protocol

**Fig. 1:**
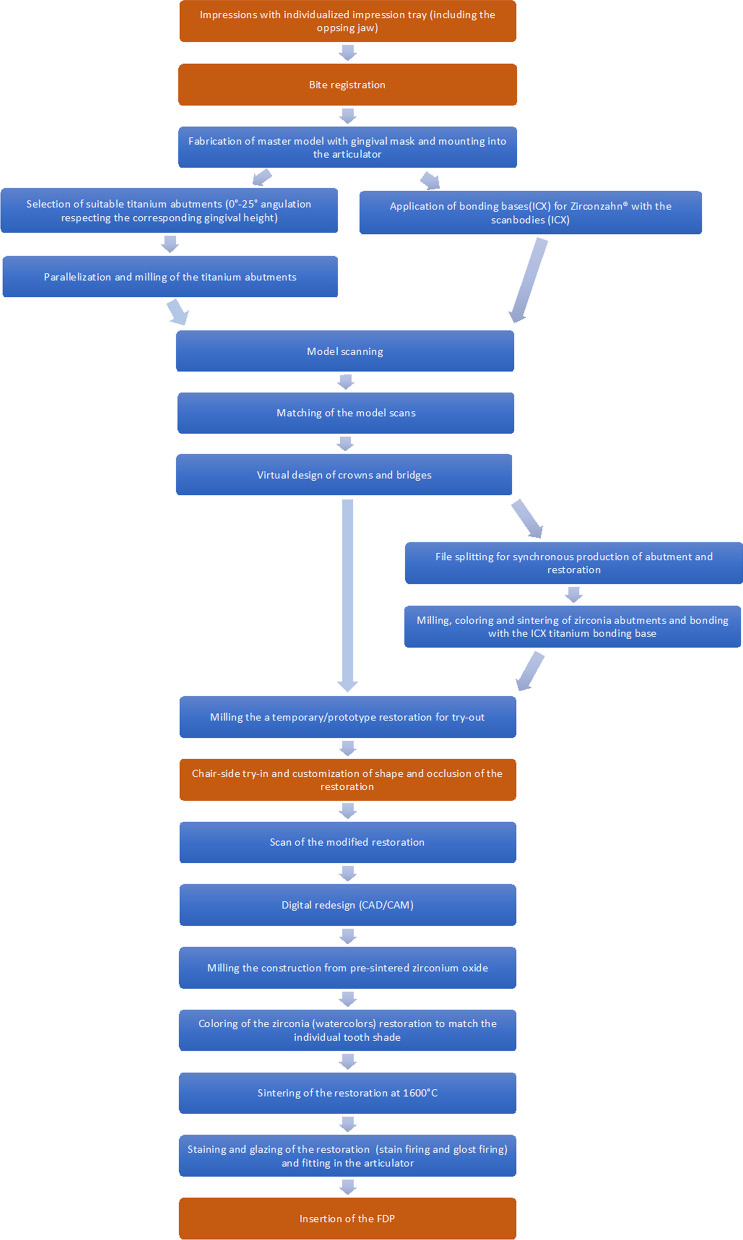
Laboratory (blue) and clinical prosthetic (brown) workflow: The procedure that applies to both abutment types can be found in the middle. Deviations from this procedure can be found on the left for titanium abutments and on the right for zirconium abutments. The scanner used in the laboratory setting was the S900 ARTI (Zirkonzahn GmbH, Gais, Italy). ICX scan bodies (medentis medical GmbH, Bad Neuenahr-Ahrweiler, Germany) and the ‘Modellier ‘ software, as well as watercolors by Zirkonzahn GmbH (Gais, Italy) were used. As synthetic material for the prototypes, Temp Basic (Zirkonzahn GmbH, Gais, Italy) PMMA material was used

The implants (ICX Premium line, medentis medical GmbH, Bad Neuenahr-Ahrweiler, Germany) were placed under local anesthesia (articaine solution with an adrenaline concentration of 1:100 000). All implants were inserted epicrestally, and patients received perioperative prophylactic antibiotic treatment with amoxicillin/clavulanic acid 875/125 mg (2x/d) for five days. Patients allergic to penicillin received clindamycin 300 mg (1–1-1). The closed healing time was up to six months in the maxilla and up to three months in the mandible. In cases that required augmentation, grafting with bone blocks (autologous bone), or maxillary sinus floor augmentation (autologous bone and xenogenic material), the closed healing period was another three months. The prosthetic superstructure of the bridges was designed with specialized prosthetic planning software (Modellier, Zirkonzahn GmbH, Gais, Italy) and manufactured from zirconium oxide (Prettau® Zirconia, Zirkonzahn GmbH, Gais, Italy). The bridges were cemented with an adhesive and self-adhesive cement (Rely X, Unicem 2, 3 M, Saint Paul, USA). The prosthetic and laboratory workflow is illustrated as in Fig. 1.

### Radiographic evaluation

Peri-implant bone levels were measured by analyzing panoramic (Orthophos S, Dentsply Sirona, Charlotte, USA) radiographs taken during recall via the methodology described by Gomez-Roman, calculating the effect of metric distortion of the radiographs [13]. Consequently, radiographs taken after implantation were analyzed retrospectively and compared to those taken at the time of the clinical evaluation. A single investigator performed the measurements as previously described and illustrated [14].

### Clinical evaluation

**Fig. 2:**
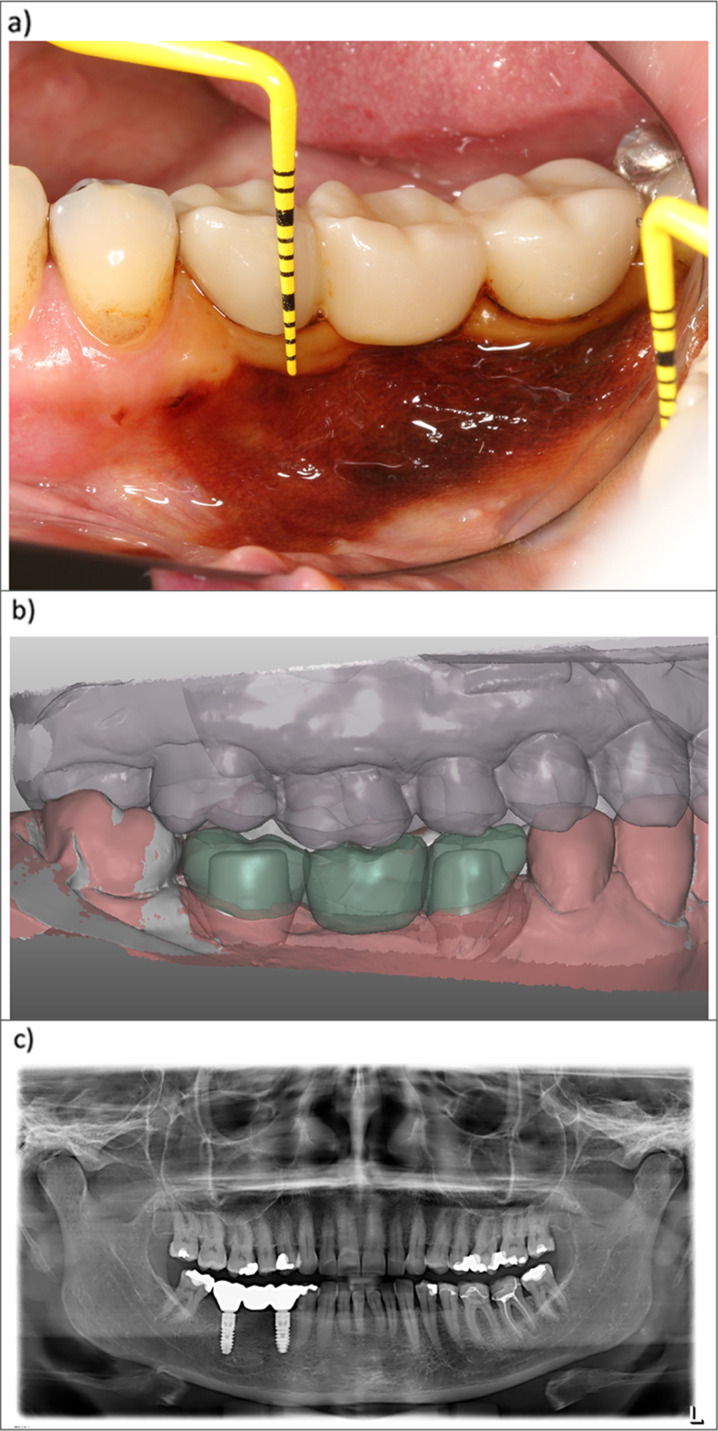
**a**–**c** Case of a zirconium hybrid abutment bridge: **a** Illustrates the measurement of the amount of keratinized gingiva using iodine solution (mirrored view) **b** demonstrates the digital implementation of the surrounding soft tissues in pink (transparent) and bone in grey visualizing the abutment structure beneath the gingiva and allowing for the measurement of the gingiva thickness.

**Fig. 3:**
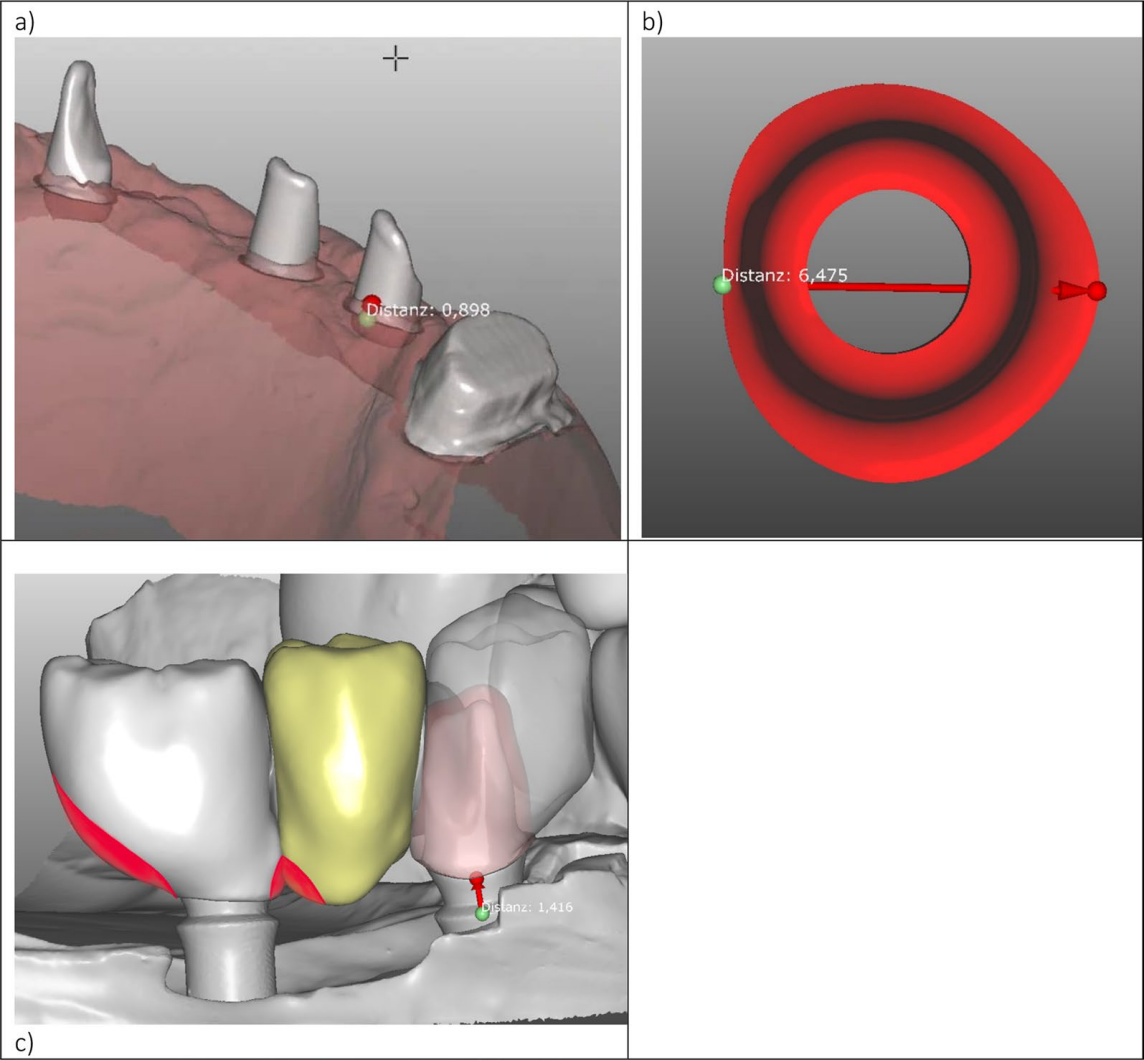
**a**–**c** Illustration of the software-based data analysis to evaluate biological a and design features of the abutment structure in the horizontal **b** and vertical **c** dimensions. Overextended portions of the crown that negatively influence the emergence angle and profile are highlighted in red furthermore, **c** illustrates an example of a titanium abutment restoration

**Fig. 4:**
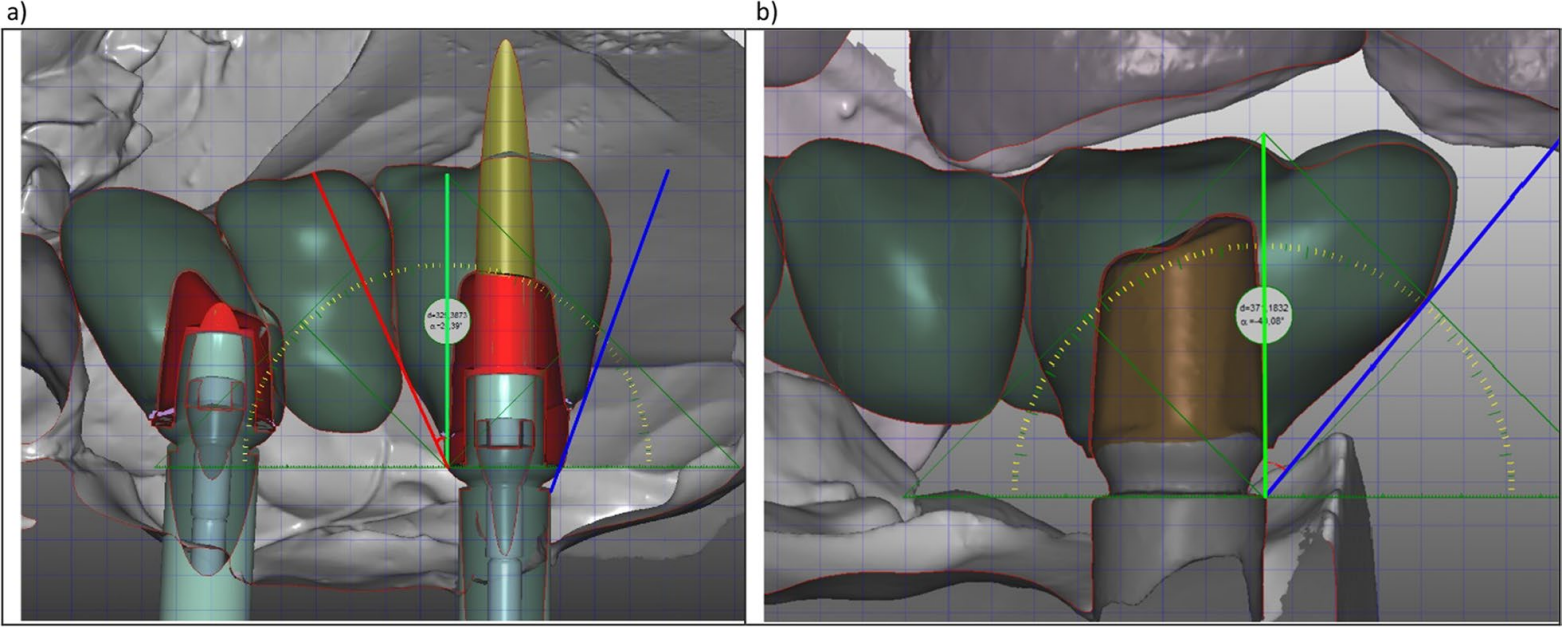
**a**, **b** Measurement of the emergence angle (the angle between a parallel to the implant axis through the interproximal border of the implant collar (green line) and the tangent to the restoration from the implant collar border (blue line) in the Zirkonzahn software (Zirkonzahn GmbH, Gais, Italy) as described by Katafuchi et al. [19] (**a** and **b**). The red line indicates the same measurement from the border of the adhesive base (AB) instead of the implant collar border (**a**). Depending on the design of the AB and crown, the blue line can cut the border of the AB. Due to the lack of an AB only one measurement was performed for the perfabricated titanium abutments (**b**).

The cross-sectional clinical evaluation was performed when patients presented for their regular implant follow-up. It included an assessment of biological as well as technical factors. For each patient, peri-implant health was measured via bleeding on probing (BoP), probing depth (PD), (modified) plaque index (mPI), visible titanium (VT), periodontal inflamed surface area (PISA), and periodontal epithelial surface area (PESA) using a standard periodontal probe (PCV 12 PT, Colorvue™ Probe, Hu-Friedy, Chicago, USA) [15–18]. Furthermore, the thickness of the peri-implant gingiva was visualized via implantology planning software (Modellier, Zirkonzahn GmbH, Gais, Italy) and the configuration of the keratinized gingiva, and phenotype around the implants were evaluated clinically and visualized via potassium iodine solution (Figs. 2 and 3). Furthermore, technical parameters such as implant length (IL), implant diameter (ID), abutment ellipse circumference (EC), abutment shell surface (SS), abutment truncated cone angle or abutment (truncated cone) angle (AA), as well as technical complications such as loosening of the abutment, chipping fractures and recementing of the crowns were evaluated, as well. The technical implant, abutment and prosthetic parameters were measured using the planning software (Modellier, Zirkonzahn GmbH, Gais, Italy) (Fig. 3). The emergence angle (EA) was measured as described by Katafuchi et al. However, the Zirkonzahn software (Zirkonzahn GmbH, Gais, Italy) was used for the respective measurements instead of interpreting the conventional dental films, as illustrated in Fig. 4. [19] The AA was calculated via the abutment height (implant shoulder-to-crown margin) and the abutment’s diameter at the implant shoulder and crown margin level.

Different case definitions of peri-implantitis were included in the analysis to improve the specificity the evaluation and account for a variety of definitions in the literature: ≥ 2 mm bone loss without BoP (bone loss without inflammation), ≥ 2 mm bone loss with one bleeding spot, ≥ 2 mm bone loss with two bleeding spots, and each category with ≥ 3 mm bone loss [20].

### Handling of correlated data and variables

For descriptive analysis relative frequencies, median, mean and standard deviations were computed. Scatterplots were used for graphical presentation.

In the case of normally distributed data, group differences were examined with linear mixed models. In the case of binary data, analysis for group differences was performed with mixed logistic models.

In both situations, the patient was considered as random effect. Due to the varying amounts of time passed since implantation in this cross-sectional setting, the yearly bone loss per implant was calculated by dividing the observed bone loss by the observation time. Due to the skewness of the distributions, the bone loss parameters were logarithmized for the statistical tests.

In the case of non-normal distribution, data were pooled on patient level and subsequent analysis of group differences was performed using the Wilcoxon rank sum test.

The statistical significance level was set at < 0.05.

All statistical analyses were performed using the STATA 17.0 program (StataCorp, College Station, Texas USA).

## Results

### Descriptive data

A total of 462 implants, all loaded with zirconium bridges, were included for evaluation. Of these, 244 implants (52.81%) in 52 patients were restored with prefabricated titanium abutments, and 218 (47.19%) in 50 patients with hybrid-zirconium abutments. Of 228 patients that were evaluated for recruitment 102 patients could be included. The mean number of implants per patient was 4.5 and the mean number of implants per FDP was 3.5. The pTiA group consisted of 23 men (44.23%) and 29 females (55.77%), whereas 17 males (34%) and 33 females (66%) could be included in the ihZiA group. Age (mean of 65.1 years in men and 63.7 years in women), smoking status (86.3% non-smokers), alcohol consumption (no consumption in 29.4%, light consumption in 51.2, moderate consumption in 8,8 and heavy consumption in in 9.8%), and diabetes (6.9% of the patients) were similarly distributed between the two groups. The mean age was 65.0 years in the pTiA group and 63.5 years in the ihZiA group (mean age overall 64.3). Four out of 52 (7.69%) patients in the pTiA group and three of 47 (6%) patients in the ihZiA group were diagnosed with diabetes mellitus (6.9% of all patients). Seven of 45 patients in the pTiA group and seven of 43 in the ihZiA group were active smokers.

Overall, 34 of the 102 patients (33.33%) had received periodontal therapy prior to implantation. Of these, 58.8% (n = 20) were in the pTiA group and 41.2% were in the ihZiA group. With 82.7% (pTiA group) and 90% (ihZiA group), the “thick” gingival phenotype was predominant.

The abutment-specific descriptive parameters are summarized in Table 1.

**Table 1:** Mean ± standard deviation in the two subgroups

Abutment type	pTiA	ihZiA	p-value
Visible titanium (mm)	0.38 ± 0.8	0.06 ± 0.3	**0.0008**
Implant length (mm)	10.56 ± 1.4	10.72 ± 1.3	0.412
Implant diameter (mm)	3.76 ± 0.2	3.80 ± 0.2	0.826
Mod. plaque index	1.07 ± 1.2	1.25 ± 1.4	0.139
Mod. bleeding index	1.29 ± 1.6	1.39 ± 1.7	0.784
Probing depth	2.72 ± 0.7	2.74 ± 0.5	0.954
Implant shoulder – crown margin (mm)	1.88 ± 0.9	2.62 ± 0.9	** < 0.0001**
Ellipse circumference (mm)	13.76 ± 1.3	19.06 ± 1.9	** < 0.0001**
Abutment shell surface	22.97 ± 11.5	45.65 ± 16.9	** < 0.0001**
Abutment truncated cone angle (°)	108.34 ± 9.7	102.32 ± 6.9	** < 0.0001**

### Outcome data

Bone resorption was not associated significantly with the amount of keratinized gingiva, the mPI, BoP or the implant shoulder-to-crown margin. Increasing PD for pTiA, on the other hand, showed significant association with higher bone resorption (p = 0.014), while this could not be observed for ihZiA (p = 0.072) (Table 2).

**Table 2:** **a**–**f** Influence of various factors on peri-implantitis according to different definitions respecting bone loss (BL) and bleeding spots

a)				
≥ 2 mm BL no bleeding	OR	95% CI		p-value
Time adj. bone loss	1.89	0.75	4.97	0.199
Abutment type	0.18	0.04	0.89	**0.0360**
Sex	1.27	0.25	6.39	0.769
Age	1.00	0.93	1.08	0.979
Jaw	0.30	0.11	0.82	**0.019**

b)				
≥ 2 mm BL + 1 bleeding spot	OR	95% CI		p-value
Time adj. bone loss	1.75	0.73	4.21	0.212
Abutment type	0.33	0.08	1.40	0.131
Sex	2.56	0.55	11.87	0.229
Age	1.02	0.95	1.10	0.513
Jaw	0.36	0.11	1.11	0.075

c)				
≥ 2 mm BL + 2 bleeding spots	OR	95% CI		p-value
Time adj. bone loss	1.27	0.51	3.20	0.609
Abutment type	0.29	0.06	1.50	0.139
Sex	2.26	0.43	11.87	0.336
Age	1.03	0.95	1.12	0.418
Jaw	0.55	0.15	2.02	0.371

d)				
≥ 3 mm BL + no bleeding	OR	95% CI		p-value
Time adj. bone loss	3.06	0.78	12.11	0.110
Abutment type	1.02	0.14	7.28	0.983
Sex	13.46	0.73	249.19	0.081
Age	1.06	0.94	1.20	0.317
Jaw	0.15	0.02	1.06	0.057

e)				
≥ 3 mm BL + 1 bleeding spot	OR	95% CI		p-value
Time adj. bone loss	2.58	1.05	6.36	**0.039**
Abutment type	1.41	0.40	4.95	0.589
Sex	5.58	0.70	44.38	0.104
Age	1.02	0.96	1.09	0.519
Jaw	0.32	0.07	1.51	0.149

f)				
≥ 3 mm BL + 2 bleeding spots	OR	95% CI		p-value
Time adj. bone loss	2.00	0.66	6.04	0.219
Abutment type	1.73	0.32	9.43	0.528
Sex	4.01	0.41	38.96	0.231
Age	1.04	0.94	1.14	0.445
Jaw	0.58	0.11	3.19	0.531

#### Bone loss and peri-implantitis

Due to the cross-sectional setting of this study, no uniform time after implantation exists. After a mean period of 5.7 years, the mean bone loss in the pTiA group was 0.74 mm (0.75 mm mesially and 0.73 mm distally). The ihZiA group’s mean observational period was 4.9 years, with a mean bone loss of 0.70 mm (0.65 mm mesially and 0.74 distally). This results in a mathematically calculated mean annual bone loss of 0.13 mm for pTiA and 0.12 mm for ihZiA. This annual bone loss was evaluated for mesial and distal sites to assess potential influencing factors, choosing the patient as a random effect (Table 3).

**Table 3:** **a**, **b** Influence of different factors on (mathematically calculated) time-adjusted/yearly bone loss (yBL) at mesial (**a**) and distal (**b**) sites

a)				
BL mesial sites	Regression coefficient	95% CI		p-value
Abutment type	− 0.14	− 0.30	0.02	0.091
Age	− 0.00	− 0.012	0.00	0.404
Sex	0.11	− 0.06	0.29	0.188
Jaw	− 0.10	− 0.23	0.02	0.106
Smoking	0.03	− 0.20	0.25	0.820
Diabetes	0.30	− 0.02	0.62	0.069

b)				
BL distal sites	Regression coefficient	95% CI		p-value
Abutment type	− 0.11	− 0.25	0.04	0.156
Age	− 0.00	− 0.01	0.01	0.704
Sex	0.19	0.03	0.35	**0.018**
Jaw	− 0.23	− 0.36	− 0.10	** < 0.001**
Smoking	0.09	− 0.12	0.29	0.418
Diabetes	0.29	− 0.01	0.58	0.055

When adjusted for time since implantation, the impact of abutment type, sex, age, and jaw on peri-implantitis in the mixed model analysis differed depending on the respective case definitions of peri-implantitis, as illustrated in Table 2. 

Gingiva phenotype had no relevant association with bone loss for either pTiA (p = 0.382) or ihZiA (p = 0.525).

In the mixed model analysis, greater gingiva thickness at the time of the prosthetic restoration was associated significantly (p = 0.002) with less annual bone loss.

#### Implant supracrestal complex

**Fig. 5:**
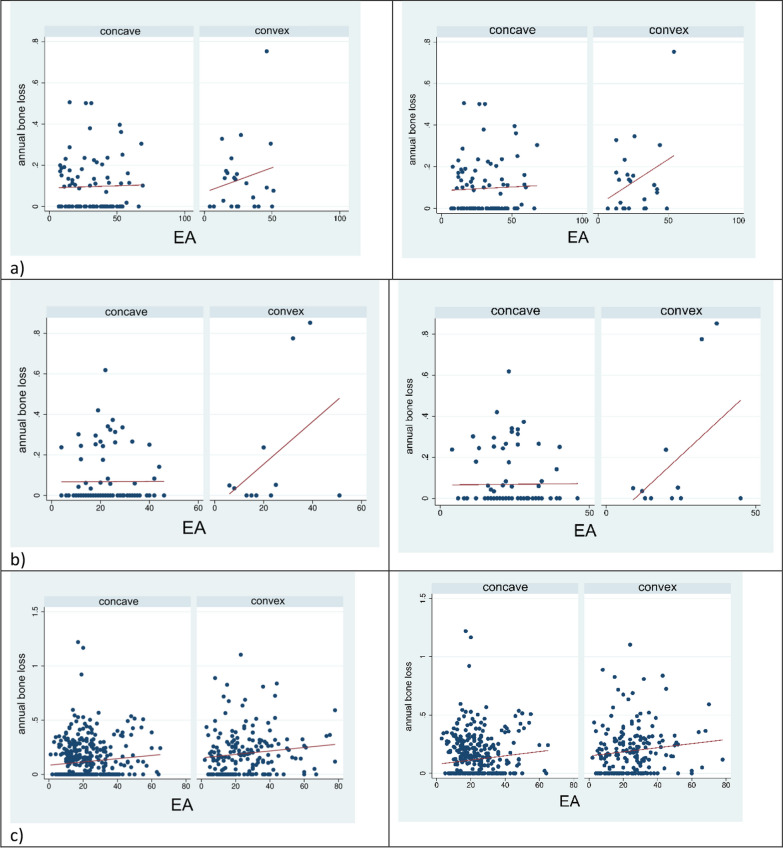
**a**–**c** Scatterplots depicting the annual bone loss in relation to the emergence profile (concave vs. convex) and emergence angle (measurements from implant collar border (left) and border of the adhesive platform (right): **a** sites at free-end position, **b** sites with neighboring tooth or implant, **c** splinted sites or facing pontics.

The evaluation of the emergence angle (EA) and the emergence profile (EP), which is defined by the contour of abutment and crown (convex vs. concave), and their impact on annual bone loss are summarized in Table 4 and Fig. 5. With 21.2° in the pTiA group and 24.3° in the ihZiA group, both demonstrated mean EA values of less than 30°. The mean EA overall (n = 924) was 23.7°. Of the pTiA sites, 82.2% (n = 401) had an EA of less than 30°. In The ihZiA group, 67.4% (n = 294) showed values in that spectrum. Of all the sites, 72.8% (n = 673) showed a concave EP in contrast to 27.2% (n = 251) with a convex EP. A concave EP was shown in 80.7% (n = 394) of the pTiA sites and 64.0% (n = 279) of the ihZiA sites.

**Table 4:** **a**, **b** Influence of emergence angle (EA) and convexity on time-adjusted bone loss. a) demonstrates the effect, if the EA is measured from the implant collar border and b) shows the effects, if EA is measured from the border of the adhesive platform. These measurements are illustrated in Fig. 4

a)			
Mesial sites	Contrast	Std. Error	P-value
EA < 30° (yes vs. no)	− 0.006	0.018	0.757
Convex (yes vs. no)	0.048	0.018	**0.009**
Combinations of EA and convexity			
> 30° and convex *vs.* > 30° and concave	0.085	0.037	0.163
< 30° and concave *vs.* > 30° and concave	0.005	0.021	0.996
< 30° and convex *vs.* > 30° and concave	0.044	0.026	0.410
< 30° and concave *vs.* > 30° and convex	− 0.078	0.034	0.137
< 30° and convex *vs.* > 30° and convex	− 0.040	0.035	0.733
< 30° and convex *vs*. < 30° and concave	0.039	0.020	0.292
Distal sites	Contrast	Std. Error	P-value
EA < 30° (yes vs. no)	− 0.019	0.018	0.279
Convex (yes vs. no)	0.034	0.019	0.067
Combinations of EA and convexity			
> 30° and convex *vs.* > 30° and concave	0.073	0.030	0.123
< 30° and concave *vs.* > 30° and concave	− 0.003	0.022	1.000
< 30° and convex *vs.* > 30° and concave	0.016	0.027	0.951
< 30° and concave *vs.* > 30° and convex	− 0.076	0.027	0.053
< 30° and convex *vs.* > 30° and convex	− 0.058	0.030	0.292
< 30° and convex *vs.* < 30° and concave	0.018	0.022	0.878
Sites with NT/I	Contrast	Std. Error	P-value
EA < 30° (yes vs. no)	− 0.023	0.033	0.477
Convex (yes vs. no)	0.094	0.043	**0.03**
Combinations of EA and convexity			
> 30° and convex *vs.* > 30° and concave	0.484	0.085	< 0.001
< 30° and concave *vs.* > 30° and concave	0.029	0.035	0.873
< 30° and convex *vs.* > 30° and concave	0.019	0.056	0.990
< 30° and concave *vs.* > 30° and convex	− 0.455	0.080	< 0.001
< 30° and convex *vs.* > 30° and convex	− 0.465	0.090	< 0.001
< 30° and convex *vs.* < 30° and concave	− 0.010	0.050	0.998
Sites at free end position	Contrast	Std. Error	P-value
EA < 30° (yes vs. no)	− 0.008	0.027	0.773
Convex (yes vs. no)	0.033	0.034	0.334
Combinations of EA and convexity			
> 30° and convex *vs.* > 30° and concave	0.049	0.055	0.855
< 30° and concave *vs.* > 30° and concave	− 0.002	0.029	1.000
< 30° and convex *vs.* > 30° and concave	0.019	0.043	0.978
< 30° and concave *vs.* > 30° and convex	− 0.050	0.055	0.836
< 30° and convex *vs.* > *30*° and convex	− 0.029	0.062	0.973
< 30° and convex *vs.* < 30° and concave	0.021	0.042	0.969
Splinted sites/facing pontics	Contrast	Std. Error	P-value
EA < 30° (yes vs. no)	− 0.026	0.016	0.102
Convex (yes vs. no)	0.031	0.015	**0.038**
Combinations of EA and convexity			
> 30° and convex *vs.* > 30° and concave	0.083	0.027	**0.028**
< 30° and concave *vs.* > 30° and concave	− 0.006	0.020	0.993
< 30° and convex *vs.* > 30° and concave	0.011	0.023	0.969
< 30° and concave *vs.* > 30° and convex	− 0.089	0.024	**0.003**
< 30° and convex *vs.* > 30° and convex	− 0.071	0.025	**0.038**
< 30° and convex *vs.* < 30° and concave	0.018	0.017	0.791

b)			
Mesial sites	Contrast	Std. Error	P-value
EA < 30° (yes vs. no)	− 0.011	0.019	0.550
Convex (yes vs. no)	0.048	0.018	**0.009**
Combinations of EA and convexity			
> 30° and convex *vs.* > 30° and concave	0.060	0.039	0.482
< 30° and concave *vs.* > 30° and concave	− 0.008	0.022	0.989
< 30° and convex *vs.* > 30° and concave	0.038	0.027	0.586
< 30° and concave *vs.* > 30° and convex	− 0.069	0.034	0.266
< 30° and convex *vs.* > 30° and convex	− 0.002	0.036	0.940
< 30° and convex *vs*. < 30° and concave	0.046	0.020	0.165
Distal sites	Contrast	Std. Error	P-value
EA < 30° (yes vs. no)	− 0.0287	0. 023	**0.031**
Convex (yes vs. no)	0.041	0.019	0.050
Combinations of EA and convexity			
> 30° and convex *vs.* > 30° and concave	0.044	0.023	0.129
< 30° and concave *vs.* > 30° and concave	− 0.026	0.028	1.000
< 30° and convex *vs.* > 30° and concave	0.008	0.030	0.902
< 30° and concave *vs.* > 30° and convex	− 0.070	0.031	0.068
< 30° and convex *vs.* > 30° and convex	− 0.036	0.032	0.350
< 30° and convex *vs.* < 30° and concave	0.034	0.032	0.817
Sites with NT/I	Contrast	Std. Error	P-value
EA < 30° (yes vs. no)	− 0.026	0.033	0.432
Convex (yes vs. no)	0.090	0.043	**0.039**
Combinations of EA and convexity			
> 30° and convex *vs.* > 30° and concave	0.481	0.085	**< 0.001**
< 30° and concave *vs.* > 30° and concave	0.026	0.036	0.912
< 30° and convex *vs.* > 30° and concave	0.017	0.056	0.993
< 30° and concave *vs.* > 30° and convex	− 0.455	0.080	**< 0.001**
< 30° and convex *vs.* > 30° and convex	− 0.465	0.090	**< 0.001**
< 30° and convex *vs.* < 30° and concave	− 0.009	0.050	0.998
Sites at free end position	Contrast	Std. Error	P-value
EA < 30° (yes vs. no)	− 0.008	0.027	0.773
Convex (yes vs. no)	0.033	0.034	0.334
Combinations of EA and convexity			
> 30° and convex *vs.* > 30° and concave	0.049	0.055	0.855
< 30° and concave *vs.* > 30° and concave	− 0.002	0.029	1.000
< 30° and convex *vs.* > 30° and concave	0.019	0.043	0.978
< 30° and concave *vs.* > 30° and convex	− 0.050	0.055	0.836
< 30° and convex *vs.* < 30° and convex	− 0.029	0.062	0.973
< 30° and convex *vs.* > 30° and concave	0.021	0.042	0.969
Splinted sites/facing pontics	Contrast	Std. Error	P-value
EA < 30° (yes vs. no)	− 0.030	0.016	0.071
Convex (yes vs. no)	0.033	0.015	**0.029**
Combinations of EA and convexity			
> 30° and convex *vs.* > 30° and concave	0.071	0.029	0.113
< 30° and concave *vs.* > 30° and concave	− 0.015	0.020	0.907
< 30° and convex *vs.* > 30° and concave	0.009	0.023	0.987
< 30° and concave *vs.* > 30° and convex	− 0.086	0.025	**0.007**
< 30° and convex *vs.* > 30° and convex	− 0.062	0.025	0.110
< 30° and convex *vs.* < 30° and concave	0.02	0.017	0.563

#### OHIP scores

**Fig. 6:**
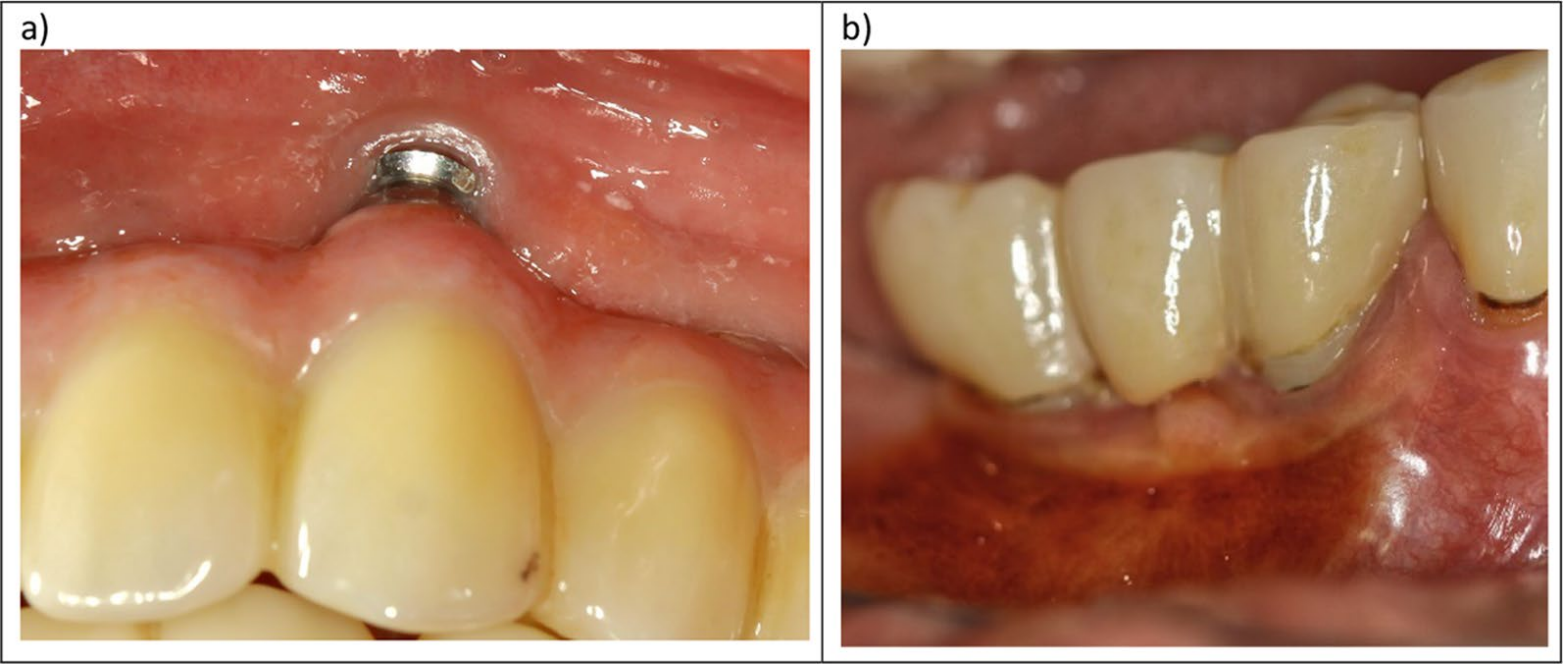
**a**, **b** Clinical view of abutment exposure of a titanium abutment region 23 **a** and two zirconium hybrid abutments in the regions 34 and 36 after iodine application **b** – view via mirror)

pTiA OHIP-G 14 levels were significantly higher if locations with exposed titanium surface were present in the visible area from the second premolar to the second premolar in both jaws (p = 0.04). In contrast, no significant difference could be observed for ihZiA (p = 0.67). The OHIP-G 49 scores were not significantly influenced by the amount of visible titanium in both groups (pTiA: p = 0.09; ihZiA: p = 0.43). pTiA in the visible area showed significantly higher exposure of titanium overall (Table 1 and Fig. 6). Abutment type alone, regarding all evaluated implants (independently from implant location), did not have a significant influence on OHIP-G 14 (p = 0.76) and 49 (p = 0.44) scores.

## Discussion

As part of the iterative process in patient care, innovations or clinical applications have to be critically analyzed. Therefore, this work aimed to investigate the outcome of individualized hybrid zirconia abutments for cemented zirconia bridges after switching from prefabricated titanium abutments. Despite many obvious reasons why this change should be beneficial for the quality of dental restorations and, consequently, patient satisfaction, it is crucial to perform critical clinical investigation and quality control.

The “implant supracrestal complex” is a recently introduced term that summarizes the implant-abutment-prosthesis complex, comprising factors such as the emergence profile, emergence angle, and implant abutment junctions [21]. But even though the authors postulated that these factors could influence the short- and long-term clinical outcome in terms of peri-implant tissue health, they could not report on corroborating evidence that the prosthetic abutment or its material (zirconia vs. titanium) had a relevant impact on the risk of peri-implantitis [22]. In the present study, design features of the two abutment types diverged significantly, such as implant shoulder-to-crown margin, ellipse circumference, shell surface, and abutment angle, as illustrated in Table 1. Individualized shapes, at least in theory, should lead to improved, more natural gingival esthetics by supporting soft tissues, maintain gingiva thickness and lead to a more favorable anatomic shape and EP. Still, in contrast to the emergence profile, none of these properties could be singled out as an individual risk factor for peri-implantitis. Whereas the abutment angle in ihZiA was significantly smaller than in the pTiA group, the shoulder-to-crown margin, ellipse circumference, and emergence profile/shell surface were significantly bigger. Nevertheless, the aim was to optimize the individual anatomical shape of the abutment, not to minimize or maximize any of these technical abutment-specific parameters.

Nevertheless, individualized abutments facilitate an advantageous concave emergence profile with an angulation tailored to the specific anatomical situation [19, 23]. This could improve accessibility for oral hygiene measures and provide an approximation to a favorable emergence angle of less than 30° [19, 23, 24]. Nevertheless, the measurements of Katafuchi et al., that lead to the recommendation of an EA of < 30°, were done on restorations without platform switching [19]. In this study on the other hand all implants were restored taking advantage of platform switching. Notably individualized ihZiA were restored with a uniformly designed adhesive base, which made measurements more difficult resulting in an initial EA of 30° from the implant collar border to the upper border of the adhesive base. For that reason, angles for the ihZiA group were measured from the adhesive base such as the implant collar border as depicted in Fig. 4. Notwithstanding the method of measurement, the impact of EA and EP was similar, when compared to ihZiA. The evaluation of the EP in this study provided further evidence that, especially in crowns emerging convexly, higher EA values are especially detrimental and lead to increased BL. To a lesser but still significant degree, this can also be stated for pontics and splinted implants and should also be considered for the design of milled bars. Due to the limited number of observations and the consideration of implant clustering in single patients, no statistically significant difference could be observed for free-end sites. Since these are explicitly located distally, only a tendency toward an impact of shape (p = 0.067) could be observed when evaluating distal sites.

Nevertheless, this fact and Fig. 4a leave room for speculation that studies with higher sample sizes of implants positioned in free-end locations might also show an impact of the EP in these situations. The analysis of different combinations of EA and EP shows that shape is essential when the EA is larger than 30° since no significant impact could be found when comparing EA smaller than 30° regarding sites with a neighboring tooth or pontic, as well as free-end positions. Therefore, the shape of individualized abutments should allow for the compensation of clinical situations that lead to higher EA. Interestingly, the relevance of the EP for splinted sites and those facing pontics was remarkable. In these cases, EA of less than 30° and with a concave shape was correlated with significantly less bone loss than EA of more than 30° and a convex profile. Of course, EA cannot be designed infinitely small, and convexity is also limited to the clinical situation. Especially in the esthetic zone, compromises like “black triangles” can hamper patient satisfaction significantly, which might be why various recommendations for pontic design exist [25, 26]. From the implant’s perspective on the other hand, the presented results lead the authors to the conclusion that the EA should be as small as possible, but at least smaller than 30°, and combined with a convex profile. In the clinical context, the EP should therefore be convex and of an EA as low as reasonably achievable, which coincides with prioritization of esthetic factors in the esthetic zone and functional/constructional parameters in the molar region [27, 28]. These suggestions are backed by the publications by Katafuchi et al. and Soulami et al. [19, 29]. Yi et al. additionally found that splinting of implants could be considered a relevant risk factor for peri-implantitis [23]. Our study also provides evidence that the mentioned parameters for EP and EA facing pontics can be regarded as relevant risk factors. A detrimental crown and pontic design with consecutive BL is illustrated in Fig. 2c, and beneficial alterations are highlighted.

In a recent review of peri-implant soft tissue phenotype modification and its impact on bone loss, Tavelli et al. argue that low supracrestal tissue height and gingiva thickness are associated with higher marginal BL [30]. This statement is backed by observations by Linkevicius as well as Berglundh & Lindhe et al. and affects implants placed at bone level in particular, and consensus exists that a peri-implant gingiva thickness of less than 2 mm coincides with early bone loss [31–34]. This is in line with the present study’s results, which demonstrate a significant correlation between gingiva thickness and BL, further underlining the necessity to evaluate this parameter in advance and consider soft tissue augmentation, which can improve peri-implant tissue health and reduce marginal BL [30, 35]. It has to be stated though that in the patient cohort examined, thinner mucosa also led to higher EA, resulting in two factors potentially augmenting the negative effect on BL.

Proper contouring of the prosthesis was shown to improve clinical outcomes for treating peri-implant mucositis, potentially preventing peri-implantitis [24]. This might explain why bone-loss without active inflammation was significantly higher in the prefabricated pTiA group when adjusted for time, even though the values for plaque or bleeding index overall did not differ significantly between the two abutment types. On explanation might be considered an abutment associated initial bone loss, since this significant difference could only be observed for bone loss without BoP of ≤ 2 mm (Table 2). The documented bone loss could also be a sign of peri-implant disease that could be controlled with regular recalls and professional cleaning. After all, the impact might be limited since abutment type was not a significant influencing factor for the other definitions of more severe peri-implantitis, especially considering BoP as an indicator for current inflammation [36]. Bone loss of ≥ 2 mm was significantly more frequent in the maxilla when compared to the mandible. This was especially the case for distal sites (p < 0.001) in the metric analysis, as demonstrated in Table 3. The literature on whether maxillary implants are generally more prone to bone level changes is inconsistent, providing evidence for both scenarios [37, 38]. The fact that especially upper distal sites were affected in the present study might indicate that accessibility to regular dental hygiene measures could be the cause. Sex, on the other hand, was found to be a significant factor only for distal sites resulting in more bone loss in men. Considering this isolated result, the clinical relevance is somewhat limited, but according to a population-based, cross-sectional study by Varela-Centelles and colleagues, being female was associated with good oral hygiene habits [39]. Still, some studies indicate higher bone resorption in women, which aside from (distal) location, further emphasizes the potential influence of dental hygiene in this study [14, 39, 40]. All the more important is an abutment design that facilitates an easily cleanable contour of the dental prosthesis. Interestingly, while PD did correlate significantly with bone resorption in pTiA, this was not the case for ihZiA, which might result from the lower bacterial adhesion to zirconia [3, 41]. Even though it is hard to account for specific anti-bacterial surface alterations, this could indicate a lower bacterial load in the peri-implant sulci [3]. Still, overall bone loss in both groups was considerably below 1 mm after roughly five to six years, which is in line with the literature. [42, 43]

To evaluate overall treatment success, the esthetic outcome and patient satisfaction also have to be considered, as they influence the patient’s QoL [44, 45]. Aside from the esthetic and natural shape of the individualized ihZiA, leading to a more natural scalloped look of the gingiva, pTiA in this study showed significantly more visible titanium (p = 0.0008). Of course, the immediate visibility of minor recessions in pTiA influences this. In restorations with ihZiA, the darkish titanium coloration, which stands in stark contrast to the ceramic crowns, becomes visible only after the recession leads to the exposure of the entire abutment. Furthermore, bone resorption did not correlate with PD to the same extent as with pTiA, potentially leading to a more stable gingival margin. While abutment type alone did not impact OHIP scores, the amount of visible titanium in the esthetic zone group significantly influenced OHIP-14 scores in the pTiA group. Taken together, this demonstrates a measurable but minor effect on patients’ QoL, considering that this was not the case for OHIP-49 scores.

Nevertheless, this emphasizes that patient expectations have risen and visible metal components are considered unacceptable, ultimately affecting patient satisfaction [45]. Figure 6 underlines this assumption. Nelson et al. and Hu et al. showed that gingival display in Caucasians can be relevant even in the first molar region [27, 28]. While this was the case for elderly patients, it was especially relevant in the younger population, who showed papillary display of over 90% in the first premolar and 85% in the second molar region [28]. Therefore, optimization of the gingival contour as well as a natural coloration without metal show is a crucial criterion for esthetically pleasing restorations, which ultimately influence the patient’s self-perception and self-confidence.

Despite the prevailing argument that ceramic restorations are more prone to chipping or fractures, none of these events occurred in this study, even though most abutments were located in the molar and premolar region known to be subjected to two to three times higher biting forces [5, 46]. This is consistent with the findings of Klongbunjit and colleagues, who found in vitro that hybrind and titanium abutments had comparable stability and strength when subjected to bending and torque fatigue tests [47]. Similarly Al-Zordk et al. did observed high fracture resistance of zirconia hybrid abutments, which exceeded the maximum masticatory forces in molar teeth by a wide margin [48]. Waltenberger and Wolfart even described a concept, that utilizes a custom-made, adhesively bonded zirconia abutment secured to a titanium base [49]. This abutment is digitally designed and placed upon implant placement, enabling immediate loading with a provisional PEEK crown [49].

A potential limitation of this study arises when comparing prefabricated with individualized abutments and is inherent in the presented setting after changing the standard of care and documenting the respective outcomes. Another limitation is the evaluation via panoramic x-ray, which bears limitations in the evaluation of bone loss in the anterior region. However, only radiographs of sufficient quality were included. Furthermore, the annual bone loss is mathematically calculated and therefore varying dynamics of tissue inflammation over time are not reflected in this figure.

According to the results of the present study, the outcome of individualized ihZiA was similar to that of pTiA. They both provide a viable option for anatomically correct and esthetically pleasing implant-based dental restorations. Furthermore, early-stage peri-implantitis was less frequently encountered in ihZiA, and OHIP-14 scores in patients of the pTiA group were significantly impacted by visible titanium. Therefore, ihZiA, if properly designed, could be superior in terms of peri-implantitis prevention and QoL. Still, further studies need to verify this assumption since the true impact of the material can be evaluated only if the same design features are applied. Therefore, this study is limited to the comparison of these specific pTiA and ihZiA in cemented zirconia FDP and the results cannot be transferred to other platforms without careful consideration and constraints. This is due to the great variety of abutment and fixation systems, which bear specific strengths, weaknesses and limits the generalizability of this study. Nevertheless, the present study highlights the clinical relevance of the supracrestal complex. Moreover, abutment features alone insufficiently reflect the interproximal situation. Thinner peri-implant soft tissue at the time of the prosthetic restoration had a significantly negative impact on annual bone loss. A convexly shaped EP should be avoided in all circumstances, and the EA should be designed as low as reasonably achievable.

## Data Availability

The datasets used and/or analysed during the current study are available from the corresponding author on reasonable request.
